# Women’s Decision-Making about PrEP for HIV Prevention in Drug Treatment Contexts

**DOI:** 10.1177/2325958219900091

**Published:** 2020-01-10

**Authors:** Yilu Qin, Carolina Price, Ronnye Rutledge, Lisa Puglisi, Lynn M. Madden, Jaimie P. Meyer

**Affiliations:** 1 Internal Medicine Primary Care Residency Program, HIV Training Track, Yale School of Medicine, New Haven, CT, USA; 2 AIDS Program, Yale School of Medicine, New Haven, CT, USA; 3 Brigham and Women’s Hospital, Boston, MA, USA; 4 Department of Medicine, Section of General Medicine, Yale School of Medicine, New Haven, CT, USA; 5 APT Foundation Inc, New Haven, CT, USA

**Keywords:** women, PrEP, HIV prevention, substance use

## Abstract

Despite pre-exposure prophylaxis’s (PrEP) efficacy for HIV prevention, uptake has been low among women with substance use disorders (SUDs) and attributed to women’s lack of awareness. In semistructured interviews with 20 women with SUD and 15 key stakeholders at drug treatment centers, we assessed PrEP awareness and health-related decision-making. Women often misestimated their own HIV risk and were not aware of PrEP as a personally relevant option. Although women possessed key decision-making skills, behavior was ultimately shaped by their level of motivation to engage in HIV prevention. Motivation was challenged by competing priorities, minimization of perceived risk, and anticipated stigma. Providers were familiar but lacked experience with PrEP and were concerned about women’s abilities to action plan in early recovery. HIV prevention for women with SUD should focus on immediately intervenable targets such as making PrEP meaningful to women and pursuing long-term systemic changes in policy and culture. Efforts can be facilitated by partnering with drug treatment centers to reach women and implement PrEP interventions.

**What Do We Already Know about This Topic?**

Women with substance use disorders (SUD) have multiple overlapping risk factors for HIV and stand to benefit from HIV prevention tools like pre-exposure prophylaxis (PrEP), but thus far uptake in this population has been suboptimal. Prior cross-sectional and qualitative surveys have revealed that women with SUD have low awareness but high acceptability of PrEP.

**How Does Your Research Contribute to the Field?**

To probe deeper into women’s low PrEP uptake and health-related decision-making, we conducted qualitative interviews with women and key stakeholders in drug treatment programs. We found important HIV risk misperceptions and competing priorities that contribute to lack of health-care engagement and would make PrEP engagement more challenging.

**What Are Your Research’s Implications toward Theory, Practice, or Policy?**

For PrEP to be successfully implemented in drug treatment for women with SUD, it must be made contextually relevant by addressing key motivating factors and reframing estimations of personal HIV risk.

## Introduction

Women with substance use disorders (SUDs) experience high HIV risk by virtue of substance use behaviors (including injecting) and overlapping sex and drug use networks. They are more likely than women without SUD to interact with the criminal justice system (CJS),^1^ become involved in sex work,^2,3^ and experience physical and sexual violence^4^—each of which independently increases HIV risk.^5-7^ Gender-specific social and structural barriers to health-care and service engagement are often overlooked in HIV prevention interventions.^8,9^

Public health campaigns to reduce HIV risk have focused on promoting condom use^10^ and safe injection practices^11^ but these methods are often not fully user-controlled and may not be feasible in the context of reduced autonomy and exposure to intimate partner violence (IPV). In contrast, pre-exposure prophylaxis (PrEP) is potentially a partner-independent, woman-controlled tool. Clinical trials^12-14^ and post hoc analyses^15^ have demonstrated that PrEP effectively prevents HIV in high-risk groups of heterosexual women and people who inject drugs when adherence is optimized. There are multiple additional ongoing and planned PrEP demonstration and implementation projects relevant to women with SUD.^16^ Yet, PrEP remains underutilized among women.^17-19^

A recent review contends that interventions to increase PrEP uptake for people who use drugs would be more effective if they were based on an adapted information–motivation–behavioral skills (IMB) theoretical framework.^20^ The IMB model of PrEP uptake asserts that, to the extent that an eligible person is informed, motivated, and has the necessary behavioral skills to initiate PrEP, they will successfully overcome obstacles to do so. We applied the IMB model to assess potential barriers and facilitators to PrEP uptake and other forms of HIV prevention among women with SUD in treatment settings. By conducting an in-depth analysis of individual, social, and structural-level barriers to PrEP uptake women with SUD, we sought to advance the public conversation about PrEP for women with SUD beyond merely increasing awareness to targeting contextually relevant barriers.^21-23^ Wherein few women are often aware of PrEP as a personal option for HIV prevention, we expanded the scope to consider how women with SUD make HIV prevention and health-related decisions in general, thereby informing future PrEP interventions by anticipating potential areas of decisional conflict.

## Methods

### Setting

The parent study, known as OPTIONS, was designed to inform, develop, and test a patient-centered decision aid about PrEP for women with SUD (registered on Clinicaltrials.gov as NCT03651453). This study was conducted at the largest drug treatment center in a mid-sized city in New England, with nearly 5000 patients on methadone annually, approximately one-third of whom are women. A full array of medications for opioid use disorder and behavioral therapies are offered across multiple sites. HIV testing is available as “opt-out” on initial intake, and PrEP is available through onsite medical providers for people who meet CDC-recommended clinical criteria.^24^

### Participant Recruitment

Women with SUD were recruited through brochures and fliers at drug treatment facilities. Treatment center staff could refer participants through a HIPAA secure Qualtrics link. A dedicated research assistant and study coordinator screened referred participants via a private study phone line for the following inclusion criteria: self-identification as female (cis- or trans-), age ≥18 years, self-reported HIV-uninfected or status unknown, and receiving treatment at the collaborating drug treatment center. Participants were excluded if they were experiencing symptoms of physiological withdrawal that could interfere with informed consent.

Stakeholders were recruited by a trained research assistant onsite at the partnering drug treatment center. Treatment center staff in any professional capacity, including physicians, nurses, social workers, counselors, case managers, medical assistants, and administrators, were eligible to participate.

### Interview Procedures

A semistructured interview protocol, based on the Ottawa Decisional Needs Assessment,^25^ incorporated questions about HIV prevention needs, PrEP awareness, perceived role of substance use in HIV risk, opportunities for HIV prevention interventions in drug treatment settings, and basic participant characteristics. The interview focused on key domains relevant to decisional needs, including factors contributing to decisional conflict, knowledge, values, and resources for support. Women were asked to reflect on options to protect themselves from HIV, hepatitis C, and other sexually transmitted infections and decisional conflict in terms of how they weigh the pros and cons of each option (see Supplementary Appendix for topic guide).

Interviews were conducted by 2 trained research assistants at research offices, in private rooms at treatment centers, or over the phone and lasted approximately an hour. All interviews were audiorecorded. Participants were compensated with a $20 gift card. Stakeholders did not receive cash compensation for participation.

### Analysis

**Figure 1.**
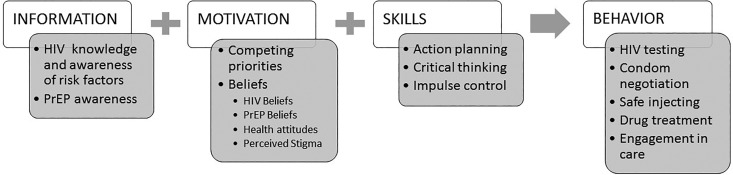
Model of HIV prevention decision-making among women with substance use disorders.

Recorded interviews were transcribed using an HIPAA-compliant transcription service and imported into Dedoose. Data were independently coded by 2 authors using predetermined nodes which were generated based on the IMB model for PrEP uptake.^20^ Through a dynamic process, findings were discussed in team coding meetings and further nodes were added or expanded to generate a hypothesized framework of health-related decision-making among women with SUD (Figure 1). *Information* was assessed in terms of knowledge about HIV and PrEP. *Motivation* was defined in terms of beliefs about HIV, HIV risk, PrEP, health (more generally), and health-care providers in relation to trust and perceived stigma. Motivation was also evaluated in the context of competing priorities, including individual-level (substance use and cravings, mental health), social (violence-exposure, commercial sex work [CSW], parenting), and structural (basic subsistence needs, criminal justice involvement) priorities. *Skills* included action planning or clarification of steps to achieve a specific goal, critical thinking or objective analysis and evaluation of an issue, and impulse control or the ability to resist an urge or impulsive behavior. The main *behavioral outcome* of interest was use of PrEP but since so few women were on PrEP, we also assessed engagement in other HIV prevention activities including HIV testing, condom negotiation with partners, safe injecting practices, and engagement in drug treatment or other medical/psychiatric care. Salient themes with exemplary quotes are presented here, organized according to the IMB model.

### Ethical Approval and Informed Consent

This study was approved by the Yale University IRB and the Operations Management Team at the APT Foundation, Inc. All eligible participants who wished to enroll were asked to complete written informed consent and a release of information. Only participants who provided informed consent completed the survey and provided data for analysis.

## Results

### Participant Characteristics

We interviewed 20 women with SUD aged between 25 and 62 years (Table 1, Panel A). Eight women reported supplemental security income as their primary source of income and none were employed at the time of interview. Most were unstably housed—12 women rented an apartment or room, 2 were staying in shelters, and the rest reported being homeless. Half of the women reported experiencing some form of physical or sexual violence in their lifetimes. Twelve women with SUD were people who injected drugs (PWIDs) and 17 had used opioids. Fourteen women identified as mothers. We interviewed 15 stakeholders (Table 1, Panel B), all of whom had direct patient contact and experience working in addiction services (ranging between 1 and 23 years). Table 2 depicts each theme with exemplary excerpts, described further below.

**Table 1.** Attributes of Participants.

(A) Target Population: Women With Substance Use Disorders					
	Age (years)	Race/Ethnicity	Education	Substance Use	Sexual Violence^a^
	42	Hispanic	Some college	PWID	Yes
	49	White	Less than high school	PWID	No
	33	White	Some college	PWID	No
	48	White	Less than high school	PWID	No
	51	White	Some college	Non-PWID	Yes
	37	White	College or more	Non-PWID	No
	52	White	High school/equivalent	PWID	Yes
	62	Hispanic	High school/equivalent	Non-PWID	Yes
	49	Black	Less than high school	Non-PWID	Yes
	62	Hispanic	Some college	Non-PWID	No
	29	White	High school/equivalent	PWID	Yes
	49	White	Less than high school	PWID	Yes
	45	White	High school/equivalent	PWID	No
	34	White	High school/equivalent	PWID	Yes
	25	Other	Less than high school	Non-PWID	No
	35	White	Less than high school	PWID	No
	41	Black	Less than high school	Non-PWID	No
	39	Native American	Some college	Non-PWID	Yes
	52	White	Unknown	Non-PWID	Yes
	36	Iranian	Some college	PWID	Yes
Summary statistics	Mean 43.5 years	10% Black	35% Less than high school	55% PWID	55% Yes
		15% Hispanic	25% High school/equivalent		
		60% White	30% Some college		
(B) Key Informants: Providers at Substance Use Treatment Facilities					
	Age (years)	Gender	Role		
	27	F	Counselor		
	61	M	Counselor		
	50	F	Counselor		
	49	M	Clinical psychologist		
	42	M	Counselor		
	35	F	Social Worker		
	31	F	Clinic director		
	42	F	Nurse		
	50	F	Counselor		
	47	F	Director		
	54	F	Personal care attendant (PCA)		
	29	F	Clinic director		
	31	M	Counselor		
	39	F	Counselor		
	64	F	Clinic director		

Abbreviations: F, female; M, male; PWID, person who injects drugs.

^a^ Sexual violence includes coercive sex.

**Table 2.** Select Themes With Exemplary Excerpts.

Information	
HIV knowledge and awareness	“…I’ve always been adamant about getting myself tested [for HIV] because I have done things that make myself at risk. I’ve been with people who I know have used intravenously. I’ve been with people who have multiple partners…if I’m using drugs…I might not remember to put on a condom…because I’m high.” (Key informant, 25-39 years)
	“I don’t think enough people know [to prevent HIV]. I think it’s like, at one point it was like when that Ryan White story was out there and everybody was talkin’ about it a lot in the late 80’s early 90’s. It was all over the place.” (Key informant, 25-39 years)
PrEP awareness	“I don’t think to do anything when I’m using. No, no one’s ever, ever told me about a medicine you could take because of the lifestyle I lead. No, I had no idea…people like me need to know that…information like this changes people’s life.” (Key informant, 25-39 years)
	“I don’t even know much about [PrEP]. If I don’t know much about it, how can I expect them [clients] to?” (Counselor)
Motivation	
Competing structural factors	
Basic subsistence needs	“It definitely kind of comes down to kind of a hierarchy of needs thing, where if housing is not stable then that kind of becomes the major decision…they’re just in survival mode a lot of times. Clients of mine that are doing sex work, that are homeless, and that are using…[HIV is] kind of low on their radar…when somebody’s living that intense of a life and then things are that difficult, it’s hard to kind of bring up anything beyond the need for getting high and finding a place to sleep.” (Counselor)
Criminal justice involvement	“Unfortunately, the judicial system really doesn’t view addiction the way it needs to be viewed. It is a medical condition, and unfortunately, part of the disease is relapse, so to continue to lock people up for exhibiting a symptom of a disease that they are not at fault for, is absurd to me.” (Key informant, 25-39 years)
Competing social factors	
Intimate partner violence exposure	“Some clients worry that…‘my significant other doesn’t even want me here [in drug treatment]. He’s not gonna want me to do that [take PrEP]. How do I explain to him that I need to be taking a medication for HIV? I can’t—he’s not gonna understand that it’s protection…He’s gonna think I’m cheating. Are you kidding me? Do you want me to get my ass beat?’” (Counselor)
Commercial sex work	“Despite me putting on my ad ‘A condom is a must’ most [clients]—I could not believe how many of them, knowing what I’m doing for a profession, wanted bareback sex…for a long time I turned down all of those offers, but…as my drug use progressed, I didn’t give a shit after a while. I really didn’t care, and all of a sudden, the prospect of making $200 to just give this guy what he wants seemed a lot more appealing than protecting my health.” (Key informant, 25-39 years)
Pregnancy and motherhood	“When you have an addiction you have to face the risk of getting pregnant and your child becoming addicted. That’s one risk. Being a woman working the streets, they worry. You get HIV” (Key informant, 40-60 years)
Competing Individual-level factors	
Meeting demands of addiction	“You don’t care about the risk. You just wanna get high…you’re not thinking about getting HIV, or if you are, you don’t really give a shit, because you’re not gonna stop what you’re doing to take precautions.” (Key informant, 40-60 years)
	“A lot of times, it’s just like the addiction comes first, and then they’re not really worried about their medical health…we do…try to promote safe sex even in recovery because a lot of times, people are not thinking about that. When we’re talking about active addiction, I think it’s difficult for a lot of people to think about preventive measures.” (Administrator)
Coping with trauma	“I’ve had a lot of sexual abuse. I was molested. I was raped…my ex set me on fire…I suffer from anxiety disorder now, bipolar and PTSD…I got addicted to the pain medication for being on it so long. Then I did go to heroin a couple times…My anxiety plays a lot in my day—everyday life, because I always have it.” (Key informant, 40-60 years)
HIV risk perceptions	“A lot of times, if people continue to perpetuate certain behaviors and they don’t see significant consequences or risks, they may continue doing it thinking that they’re invincible.” (Administrator)
	“I’d be lying if I told you I thought about it. I don’t. It’s easier not to ‘cause I live a very dangerous lifestyle. Easier just not to think of that shit…I was scared as shit when I went to the [sober] house and they gave me that quick little HIV test ‘cause really I didn’t know. I had no idea. I never thought about it.” (Key informant, 25-39 years)
PrEP beliefs and perceived stigma	“If anybody knows what [PrEP] is, and they see me with it, and wondering what I’m doing with it…They might get suspicious as to what’s up with me.” (Key informant, 60+ years)
	“I don’t mean to be moral, I just feel sex should have a little bit of thought to it. When you take a pill, it makes you feel like you don’t necessarily need to think at all about the dangers of it [sex]…. someone taking that is thinking, oh, I can’t get HIV, but they’re not thinking I could get all of these other things.” (Counselor)
Health attitudes	“What I would want from my healthcare provider was not to feel like I’m dirty or like I’m just some scumbag. I’ll be treated like everybody else whether I’m a drug addict or not…once you become a drug addict on paper, God forbid you ever need help again. You’re faking. You’re drug-seeking…I won’t go to the doctor because of those things.” (Key informant, 25-39 years old)
	“…A lot of our clients would rather go without their methadone than tell the doctor treating them that they’re on methadone…In general, I think a lot of our clients do not trust healthcare professionals. I think they trust their medical judgement, but they don’t trust that they’ll be treated with respect.” (Counselor)
Behavioral skills	
Action planning	“The way that I managed to not contract [HIV] was, there was a needle truck that was in the area that I was using in, and they were there on certain days at certain times, and I made sure to get my ass to that truck.” (Key informant, 25-39 years old)
	“One of the difficult things I think for patients, sometimes, with something like PrEP, is the idea of planning ahead, that it’s not necessarily in the moment…Whereas I think if you’re offering something around, oh, I can get you situated in terms of your housing. That’s immediate.” (Clinician)
Critical thinking skills and health literacy	“I think some people may feel overwhelmed thinking about [HIV prevention]. They may not really know how to protect themselves. They may not really know who to ask, as far as providers, what kind of information they could receive.” (Social worker)
	“I find it hard to make a decision if I don’t know all my options out there…I think the more options you’re given, the better decision you can make…I’m lucky that I moved here and have the options that I do now to get on my feet, to get off the drugs, and hopefully I’ll be a productive member of society one day, but you have to be able to have the options and education to do that.” (Key informant, 25-39 years)
Impulse control	“Once you’re smoking, your mindset is not right, so you’re liable to have sex with somebody for money, just do the things that you wouldn’t necessarily do…Mostly anybody that I got high with is dead from AIDS or getting shot or ODing. It’s crazy.” (Key informant, 40-60 years)

Abbreviation: PrEP, pre-exposure prophylaxis.

### Information

Participants generally understood basic principles of HIV transmission and treatment. Nine women reported that they underwent HIV testing frequently. Some women talked about how knowing their sexual partner, and their testing history was important for self-protection and harm reduction. Seven women reported that were abstinent. Women with friends or family with HIV were highly knowledgeable about treatment and prognosis but a recurring worry expressed across multiple interviews was that general awareness about HIV had waned in recent years.

Most women interviewed had not heard of PrEP. Among the 7 who had, 1 had learned about PrEP through a research study while the rest were through word-of-mouth. Some providers felt concerned about the low level of PrEP awareness among their clients and recommended increasing visibility through TV commercials, pamphlets, and targeted marketing ads.

Although many providers had heard of PrEP, most expressed an interest in receiving more training to better counsel clients. Some saw themselves more as “gateway providers” who could refer patients out to specialists for PrEP counseling and initiation. One provider said she raised PrEP with her male or transgender clients who had sex with men but had not considered it for heterosexual women.

### Motivation

Women’s motivation to engage in health-promoting behaviors was influenced by multiple competing priorities and certain preformed beliefs. One counselor commented: “Helping people make decisions can be tricky…you really can’t ignore the systems that people are involved with and their histories as far as the impact of trauma, poverty, mental health and how all of those intersect and really can make things difficult for people.” Women often had to make calculated tradeoffs between basic subsistence needs (food, housing, income, transportation), responsibilities of motherhood, meeting the demands of addiction, and coping with trauma. In this way, health-promoting behaviors including HIV prevention (and potentially, PrEP) were sometimes deprioritized.

#### Competing structural factors

Both women and providers noted that basic survival needs took priority over HIV prevention or health. One woman described how overcoming homelessness allowed her to gain autonomy in sexual decision-making: “I went through years back and forth of homelessness…livin’ in shelters…sleepin’ in streets, sleepin’ with people just to have a place to stay…[now] if I decide to do something we gotta have condoms on, lights on, I wanna see it, I wanna smell it, I wanna look at it.” One provider noted that his clients were being pushed out of urban centers by gentrification, thereby isolating them from resources “and having them connected to HIV prevention is almost impossible when they can’t even get to the clinic daily because their transportation is so irregular.” Beyond transportation, most women’s concerns about PrEP were logistical, including identifying and accessing a PrEP provider, having insurance to adequately cover PrEP, and being able to afford the cost of copays and associated doctor visits for follow-up care.

Stigma and fear of criminalization discouraged women with SUDs from seeking treatment and accessing health-related resources, which further contributed to isolation and generated additional logistical challenges. This isolation would likely extend to PrEP care engagement. In discussing their perceived HIV risk and prior health-care engagement, 10 women raised their prior interactions with the CJS. Seven had been previously arrested or incarcerated for drug-related offenses or prostitution, and one was on probation for prescription tampering. Preventative services and drug treatment were felt to be lacking in prisons and jails and the punitive handling of addiction by the CJS created undue burden (frequent court dates, urine drug screens, law enforcement mandates). Justice-involved women felt stigmatized and restricted: “I’m a licensed EMT. I can’t get a job anywhere cause of my criminal record.” Unemployment and financial insecurity forced some women to rely on partners for basic subsistence needs. Providers noted that, for many women, partners controlled living arrangements, household finances, and any outside communication. This may have extended to health-care engagement.

#### Competing social factors

Women described interpersonal relationships that often detracted from health-promoting behaviors, including using condoms for HIV prevention. This suggests the importance of a woman-controlled, partner-independent HIV prevention tool like PrEP. Many of the women interviewed had lifetime experiences of physical and sexual violence that forced them to choose between personal health and safety. One woman described how condom negotiation precipitated violence: “My ex…I know he slept around…when I’d say something to him, like, ‘Wear protection’ or something, he’d give me a beating thinking I didn’t trust him.” Another woman shared that her partner initiated her to injecting heroin and often secondarily injected her without her consent.

Four women who engaged in CSW tried to use condoms but felt pressured to comply with client requests because men paid more for condomless sex. Violence from commercial partners was common in CSW: “With the prostitution stuff…first you gotta not be murdered, and then you can worry about condoms.”

Pregnancy prevention was a much stronger behavioral motivator for condom use than HIV prevention, as one clinician explained: “Typically the women I’ve treated have been more focused on, ‘Is my partner gonna be wearing a condom?’ Or, ‘I don’t wanna become pregnant’ rather than, ‘Oh maybe I’m at risk of HIV.’” Until dual HIV and pregnancy prevention modalities are developed, PrEP will not be able to address women’s needs for pregnancy prevention.

Providers felt that social connections empowered clients to make healthier decisions. Five stakeholders suggested community outreach and peer support for HIV prevention: “I feel like the community does need more education on how to treat people who have an addiction or HIV, and how to give that information in a way that doesn’t make them feel condemned or shameful, like in a way that motivates them to change…If there were peer support that might help.” Women were also interested in peer-driven knowledge: “I’m gonna tell everybody I know about this medicine [PrEP]…information like this changes people’s life.”

#### Competing individual-level factors

Women’s HIV risk was primarily driven by substance use. Many women provided vivid descriptions of how getting high was, at times, the “sole and primary focus” of their lives. The need to avoid withdrawal superseded all other priorities, including personal health and safety: “I didn’t really think about [HIV risk] because it didn’t really matter. I needed to get high. I was getting high regardless, even if you told me you had AIDS. If I was sick and needed to get high and you had a needle that I had to use, I’d still use it.” Women experiencing active withdrawal also took more risks while engaging in CSW: “If your body’s sick from something, heroin or whatever, you’re not thinking about does so-and-so have a condom…You’re just gonna go, I need this money to get myself well. I’ll worry about that risk later.” PrEP programs need to consider that women at highest risk for HIV (and most in need of PrEP) might require extra support to engage.

Many women experienced lifetime trauma and 2 women discussed using drugs to cope with trauma. Providers noted that trauma reduced autonomy, self-efficacy, and self-esteem, which made it difficult for women to advocate for themselves with partners or health-care providers, including using condoms, safe injecting techniques, or PrEP to prevent HIV.

Although many women were broadly aware of HIV, most did not feel they were personally at risk despite past unprotected sex with unknown or multiple partners, engaging in transactional sex, having sex while intoxicated, or sharing injecting equipment. Two women specifically cited concerns about HIV as a reason for never sharing needles. Providers felt that women with SUD were less concerned about HIV than other health issues: “They’re more focused on the pregnancy prevention and thinking about condoms more than anything else…I don’t hear a lot of talk of [HIV]…there’s a lot more talk about Hep C just because it’s so prevalent in our population.” Many women believed that sharing needles or having unprotected sex was safer if it was with a known partner, even if the partner was engaging in high risk injecting or had HIV: “I thought, you know, it’s just me and him. He’s clean. I’ve been with him for such and such time…I’m not gonna catch HIV or anything like that from him because if I was going to catch anything, I would have already caught it.” Providers tried to help women reshape their perceptions of risk by challenging statements of denial or minimization of risk.

Five providers were concerned about some clients’ false sense of security or “invincibility” if they had averted HIV despite high-risk behaviors, subscribing to the notion that “it won’t happen to me” or underestimating their own behaviors. Some providers felt that risk misperception was not due to a deficit of knowledge but rather rationalization: “If you try and get them to do something different, you’re taking away that choice…there’s some function to the behavior that’s not immediately obvious to other people.” Many observed that clients regretted their risky decisions, but developed thought patterns, such as “if I don’t get tested then I don’t have HIV” or “if I don’t think about [HIV risk] then it’s not real and it’s not gonna happen.”

Most women who had not heard about PrEP were receptive to the idea and felt that it would be useful for women with SUD: “At the end of the day it comes down to just staying protected…We’re not gonna stop sharing needles…but knowing you can take a pill every day—using protection when we can—if we do have to use somebody’s needle, then we know we’re still gonna be okay.” Other women expressed enthusiasm about PrEP in terms of it preventing HIV but needed more information before deciding if it was right for them.

Four women and 1 provider cited concerns about risk compensation. One woman stated that the fear of HIV made her take “more precautions about other things and [HIV is] certainly not the only STD to be worried about.” PrEP stigma was also a concern, both internalized (the implications of taking PrEP in terms of the kind of person they were) and externalized (what others and partners would think of them for taking it). Providers expressed concerns that PrEP might lead to partner retaliation and IPV exposure risk.

Since PrEP is only currently available by prescription, we assessed women’s interactions with systems of care. Reluctance to interact with medical systems was largely due to perceived stigma and past experiences. Five mentioned negative past experiences with health-care providers but 2 said they would still listen to the recommendations of a trusted provider. Many stakeholders also discussed how incorrect assumptions (ie, PrEP being mistaken for HIV treatment) perpetuated stigma and resulted in negative experiences for clients. In contrast, as 1 clinical supervisor elaborated: “Having people encounter as many positive experiences with healthcare as possible, I think is really important too in helping make those decisions easier.”

### Behavioral Skills

Many women demonstrated skills to plan out health-promoting actions that are necessary for engagement in PrEP and other HIV prevention services, such as managing doctor’s appointments, navigating medical insurance, obtaining sterile injecting equipment, and completing HIV testing. One provider noted that even in active addiction, some women manage to practice safe behaviors: “There are people that are emphatic about well, whether I’m using or not, I’m gonna keep myself safe.” All 8 women who had ever tested for HIV reported being frequent testers. Women who injected drugs described obtaining sterile injecting equipment from doctor’s offices, needle syringe programs, pharmacies, and family members with diabetic supplies. Some counselors tried to work on building condom negotiation skills but recognized that power dynamics were often unfavorable.

Four women said they did not like condoms because they detracted from excitement and diminished pleasure. Planning ahead for sex was seen as “no fun” and a provider concluded: “For some women and men, I think the idea of being intimate or having sex involves spontaneity…The idea of having a lot of forethought and planning…somewhat takes the fun out of the whole situation.” Other women did think planning ahead was a part of healthy relationships, and one woman told her partner, “I’m not gonna be intimate with you until you actually go to the doctor, get the [HIV testing] paperwork, just like I did.” This is particularly relevant to PrEP which, though requiring action planning to obtain and adhere to a daily medication, does not require planning for sex.

Women also demonstrated critical thinking skills when asked to describe their decision-making processes for various health-related behaviors, such as whether to start a new medication, which is particularly relevant to PrEP initiation. For example: “I think about the pros and the cons. I read the paperwork that comes along with the medication, and I look at the side effects. Then I see how my body adjusts. If I don’t like the side effects, then I’m gonna go in [to] whoever prescribed it to me. If [side effects are] too much to deal with, then I’m gonna say, ‘Is there another drug you can give me?’ or, ‘Maybe I don’t need it.’” Both providers and patients mentioned information overload as a barrier to health-related decision-making that was especially challenging for women with low-health literacy. Eight women listed coping with side effects as the primary concern about starting a new medication, and women weighted side effects in terms of perceived severity. For example, headaches might be manageable whereas side effects “like going through chemo” would be intolerable for any medication, though not necessarily relevant to PrEP.

Women were heterogeneous in their ability to action plan and control impulses. Six providers expressed concerns about clients realistically engaging in long-term goal-directed behavior, especially during early stages of recovery. Women also identified that lack of impulse control contributed to their risk behaviors related to substance use (Table 2). Although some PrEP providers may see this as a reason to defer PrEP in women with SUD, 2 providers suggested incentivizing PrEP with immediate rewards to increase uptake: “One of the difficult things I think for patients, sometimes, with something like PrEP, is the idea of planning ahead, that it’s not necessarily in the moment…Whereas I think if you’re offering something around, oh, I can get you situated in terms of your housing. That’s immediate.”

## Discussion

In this qualitative study, we explored why and how women with SUD, a key target population for HIV prevention, have low awareness but high potential acceptability of PrEP and other HIV prevention tools. Qualitative findings illustrate how a combination of information, motivation, and behavioral skills are necessary to engage in health-promoting behaviors in general and PrEP specifically (Figure 1). Decision-making practices around health were driven by competing priorities, health beliefs, and health attitudes. This deep dive into women’s decision-making processes and choice heuristics is critical to developing and implementing effective multilevel interventions to increase PrEP uptake among women with SUD.

Although stakeholders acknowledged that limited direct-to-consumer marketing and lack of inclusive messaging affected women’s PrEP awareness, other issues shaped women’s health-related decision-making more broadly. Women consistently underestimated personal risk for HIV so that PrEP, when pitched as an HIV “risk reduction tool,” was not personally relevant. Risk misperception among women with SUD seemed to have resulted from rationalization and minimization of risk rather than from knowledge gaps. Women tended to appraise HIV risk in ways that ultimately supported the conclusion they desired, which has been described in other at-risk populations,^26^ and simplifies complex HIV risk estimation into rules that conflate familiarity with trust and safety (eg, known partners are safe partners, monogamous sex is safe sex) or ascribe absolute predictive value to social indicators (eg, people who are married or monogamous are not at risk for HIV). In our study, women selectively focused on partner familiarity while minimizing partners’ risk. This unconscious cognitive bias is particularly problematic for women because current risk assessments and PrEP eligibility criteria require women to appraise their partners’ behaviors (eg, whether they also have sex with men or inject drugs).^27^ One benefit of PrEP over other HIV prevention tools (like condoms) is that it is effective regardless of personal or partners’ type of risk behaviors.

Some women described personal “invincibility” or deliberately avoided HIV testing despite high-risk exposures. For many women, this compounded impulsivity and difficulties with long-term planning, which has been observed in other studies of individuals with SUDs^28^ and people with behavioral addictions like gambling.^29^ Mindfulness-based interventions and goal management training may improve executive function and realign risk perceptions among people with SUDs.^30,31^ Given the high prevalence of violence, trauma, and post-traumatic stress disorder (PTSD) among women with SUD,^1,8,9^ 2 upcoming trials are adapting mindfulness-based interventions for women with SUD with a history of trauma.^32,33^ These interventions may also be effective at increasing PrEP uptake or engagement in harm reduction programs, though further research is needed.

Women with SUD in this study often struggled to meet basic subsistence needs such as housing, transportation, medical care, and a source of stable legal income. These competing priorities may have decreased motivation to engage in HIV prevention. Other large studies, including HPTN 064,^34^ have shown how poverty, food insecurity, and ongoing substance use contribute to disparate HIV incidence rates. For PrEP to be meaningful to the women who need it most, it needs to be part of a program (not simply a drug) that includes wraparound services that improve the quality of their daily lives, like housing, employment assistance, and vocational training.

Other social determinants of health played a key role in women’s health behaviors related to PrEP and HIV prevention. The prevalence of lifetime gender-based violence exposure among US women is 36%^35^ and 2 to 5 times higher among women with SUD.^4^ Women with SUD experience excess mortality due to violence compared to age-matched peers and to men who use drugs.^6^ Previous studies have shown a direct correlation between violence and HIV risk,^1,2^ the confluence of which among women with SUD is known as the substance abuse, violence, and AIDs syndemic.^4,36^ Women with SUD have high rates of PTSD,^1^ which can reduce autonomy, self-efficacy, and self-esteem. Moreover, women with SUD, particularly those who exchange sex, often have limited social capital to negotiate condom use or advocate for personal health and safety. Consistent with findings from other studies, women engaging in sex work in this study reported financial incentives for unprotected sex.^9^ Economic dependence on partners is a strong and consistent predictor of condomless sex.^37,38^ In contrast, PrEP is a user-controlled tool that does not depend on favorable power dynamics. Other approaches to increase women’s empowerment include microfinance interventions,^34^ which have focused on building economic skills and generating an independent source of income.

Moving forward, strategies to increase PrEP uptake for women with SUD include incorporating contextually relevant messaging. For instance, PrEP messaging campaigns need to be mindful of stigma,^39^ which interferes with service engagement and increases HIV risk. Negative stereotypes about women with SUD reinforce internalized stigma and foster poor self-efficacy, which only further reduces women’s agency to engage in health-promoting behaviors. Perceived stigma from partners, the local community, and society at-large makes women less likely to initiate PrEP and deters them from engaging with health-care systems. Effective strategies to integrate HIV prevention into drug treatment programs must take these realities into account so that women do not have to choose between seeking SUD treatment and other priorities, such as childcare.

Providers in our study were concerned about overwhelming women if they introduced PrEP during early recovery and treatment engagement. Overwhelming messaging may compound women’s mistrust in providers or health systems, rendering providers potentially less effective messengers about PrEP than peer (or “socially concordant”) educators. Extant preliminary data on PrEP peer navigators are among men who have sex with men and further research is needed on PrEP peer navigators for women.^40^

Providers also identified that women’s lack of action planning and impulse control could be major impediments to health-care engagement and medication adherence. This is especially relevant to PrEP uptake and adherence when most current formulations of PrEP are delivered as a once-daily medication. Because of lower concentrations of tenofovir in vaginal or cervical (as opposed to rectal) tissues, women require higher levels of PrEP adherence to achieve similar protective benefit. Underestimations of women’s potential to adhere may bias providers against providing PrEP to women with SUD. Similar biases are pervasive in HIV treatment, leading clinicians to defer antiretroviral therapy for people with SUD living with HIV and resulting in increased HIV-related morbidity and mortality.^41^ Yet studies from HIV treatment have shown that people with SUD are able to adhere to medications and successfully achieve similar clinical outcomes as people without SUD when appropriate support, including drug treatment, is provided.^42^ The same should extend to PrEP.

This study is the first, to our knowledge, to qualitatively assess PrEP awareness among women with SUD and consider drug treatment centers as potential sites for PrEP outreach and dissemination for women. Findings may not be generalizable to other geographic settings, although qualitative studies generally aim instead for depth of experience. Some of our participants were older than most patients with PrEP and had had prolonged experience with SUD treatment. Because participants were aware that these interviews were part of an HIV prevention research project, there may have been some selection or reporting bias. All participants were either in treatment or affiliated with a single-drug treatment provider and may differ from other women with SUD who are not currently in drug treatment.

## Conclusion

Pre-exposure prophylaxis is a highly effective evidence-based HIV prevention tool for women with SUD who may lack social capital to negotiate condoms. HIV prevention is not solved alone with a PrEP prescription and housing support, mental health care, domestic violence resources, and accessible childcare are needed in addition to PrEP to comprehensively address the multiple contextual factors that increase women’s risk for HIV and prevent engagement in prevention services. Fully integrating PrEP into drug treatment settings is key for reaching women with SUD.

## Supplemental Material

Supplemental Material, Appendix_Protocol_for_Target_Population_1.19.18 - Women’s Decision-Making about PrEP for HIV Prevention in Drug Treatment Contexts Click here for additional data file. Supplemental Material, Appendix_Protocol_for_Target_Population_1.19.18 for Women’s Decision-Making about PrEP for HIV Prevention in Drug Treatment Contexts by Yilu Qin, Carolina Price, Ronnye Rutledge, Lisa Puglisi, Lynn M. Madden and Jaimie P. Meyer in Journal of the International Association of Providers of AIDS Care (JIAPAC)
