# Supplementary material for: Women’s Decision-Making about PrEP for HIV Prevention in Drug Treatment Contexts
Source: J Int Assoc Provid AIDS Care. 2020 Jan 10;19:2325958219900091. doi: 10.1177/2325958219900091 (PMC7099671; doi:10.1177/2325958219900091)
Supplement: Supplemental Material, Appendix_Protocol_for_Target_Population_1.19.18 - Women’s Decision-Making about PrEP for HIV Prevention in Drug Treatment Contexts [file Appendix_Protocol_for_Target_Population_1.19.18.pdf]

Decisional Needs Assessment: Topic Guide for Target Population

Good morning/afternoon/evening. My name is ____________ and I work at the Yale School of Medicine. I am going to interview you for a research project called OPTIONS, which we are doing with both healthcare workers and people who receive healthcare, to learn more about the needs people have when making decisions about protecting themselves from HIV and Hepatitis C. We want to better understand people's behaviors so that we can help find ways to protect people against HIV.

During this interview, I will be asking you some questions about the health decisions you may have made as this information will help us to better understand what support is needed to help other people make decisions, too.

All of the information we collect in this interview will be kept strictly confidential. I will not mention your name during the recording, and any names that you mention during the interview will not be written out when we go to transcribe this interview. This will take about 30-60 minutes to complete, and we really appreciate your willingness to help.

1. **I'm interested in learning about your experiences with APT.** What kind of treatment do you get there? How long have you been in treatment?
2. **When did you realize that drugs or alcohol was going to be a problem for you?** *Prompt: how old, when did it start, etc.*
    - What is your primary drug of choice?

3. **What are your goals in terms of your health?** *Prompt: Do you have any goals regarding drug use or alcohol use?*

4. **Tell me about a recent situation when you used drugs**. Looking for details- who, where, what, when, how.
    - Did you think you were at risk for HIV during that time? How/why or why not?
        - If yes, what did you do about this risk?

5. **Tell me about a recent situation when you had sex**. (*Looking for details- who, where, what, when, how). Prompts: Did you use protection, did either of you use drugs during or before you had sex*
    - Did you think you were at risk for HIV at that time? How/why?
        - If so, what did you do about this risk?

6. **When you think about HIV, what sort of things come to mind? Do you ever worry about your HIV risk?**

7. **Tell me what you know about PrEP.**
    - Have you thought about PrEP as an option?

- What does your doctor say about PrEP?

RISK BEHAVIORS

8. **Can you think about any recent situations that might put you at risk for HIV?** Sexually? Related to drug use?
    - Can you think about situations in the past that might have put you at risk for HIV?
    - What is about those situations that adds risk?

9. **When you think about taking sexual risks, what sort of things come to mind?**

10. **When you think about the risks of drug use, what sort of things come to mind?**

11. **Do you usually plan ahead in terms of reducing your risk?**
    - Thinking about your most recent time using drugs or having sex, did you plan ahead in some way to reduce your risk of acquiring HIV or Hepatitis C?

DECISION

12. **What do you think are the most important decisions that women with addictions face?** *Prompt: Decisions regarding health or staying healthy/safe, housing, children, etc*

Let's focus on one particular decision, the decision about protecting yourself from HIV, Hepatitis C, and other sexually transmitted infections.

13. **Thinking about this decision, what are the options that you have to protect yourself against HIV or Hep C?** *(Probe to see if HIV pre-exposure prophylaxis, or PrEP, is one of the options they might list)*
    - What do you see as the pros and cons of each of those options? (main advantages/benefits and disadvantages/risks)

14. **Let's talk about the difficulty with making personal decisions about HIV prevention. How do/did you feel when making a decision for yourself**? *Prompt: "Sometimes people can feel unsure about what to do' or "pretty confident in their decision making" where do you fall on this spectrum.*

    [Probe behavioural manifestations of decisional conflict]

    Do you feel:

    ☐ unsure about what to do?
    ☐ worried what could go wrong
    ☐ distressed or upset
    ☐ constantly thinking about the decision
    ☐ wavering between choices or changing your mind
    ☐ delaying the decision
    ☐ questioning what is important to them
    ☐ feeling physically stressed, tense muscles, racing heartbeat, difficulty sleeping]

15. **Sometimes there can be barriers in the way of making a healthcare decisions. What things make healthcare decisions difficult for you?**

    Prompts:

    ☐ Feeling like you don't know enough about options, benefits, risks
    ☐ Lacking information on the chances of benefits and harms
    ☐ Confused from getting too much information at once
    ☐ Unclear about what is important to them
    ☐ Feeling unsupported in decision making
    ☐ Feeling pressure from others
    ☐ Not motivated or not feeling ready to make a decision
    ☐ Feeling like you don't have the ability or skill to make a decision
    ☐ Feeling these decisions aren't relevant to you or your needs

16. **Who else may be involved in making this decision with you?** *Prompt: partners, friends, parent, etc*
    - Do you ever talk to your healthcare provider about these decisions? Do you ever talk about it with your drug counselor?
      [Probe role in decision making]
      When do you have to make a healthcare decision (like taking a particular medication) with your healthcare provider, do they usually:
      ☐ Make the decision for you
      ☐ Share the decision with you
      ☐ Provide support or advice for patients to make the decision on their own

17. **What are the steps that you take in order to make a decision about whether or not to take a particular medication.**
    [Probe decision making behaviour:]
    Do you:
    ☐ Get information on options
    ☐ Get information on the chances of benefits and risks
    ☐ Consider the personal importance of the benefits and risks
    ☐ Get information on how others go about deciding
    ☐ Get support from others
    ☐ Find ways to handle pressure

18. **Is there anything else that could help you make a healthcare decision?**
    - What would hinder you (get in the way of) making this decision?
    - Do you think drugs or alcohol get in the way of you making decisions about HIV prevention? If so, how?

19. **Is there anything else that would help overcome these barriers to decision making?**

20. I will go through a list of things that might be useful for people in helping them make a decision that is right for them, which ones do you think might be useful to you?

□ Counseling from a health practitioner→ IF YES, specify what types

□ Discussion groups of people facing the same decision→ IF YES, specify what type of organization or group

□ Information materials→ IF YES, specify content

□ Health condition
□ Options
□ Benefits
□ Risks
□ Probabilities of benefits/risks
□ Help considering the personal importance of benefits versus risks
□ Guidance in the steps of deliberation and communication
□ Other, specify

IF YES, specify format

□ Booklet, pamphlets
□ Internet
□ Videos/DVDs
□ Other, specify _____________

Before we end this interview, I just have a few demographic questions:

1. What is your age?

2. What was your sex at birth?

3. What is your race/ethnicity?

4. What is the highest level of formal education you completed?

5. What is your relationship status? (married, divorced, separated, widowed, in a relationship and not married, not in a relationship)

6. What is your employment status? (employed full-time, employed part-time, not employed)

7. What is your housing status? (permanently housed, temporarily housed, shelter, homeless or sleeping in a public place)

[THANK RESPONDENT]
